# A High-Multiplicity Baculovirus Method Enables Efficient Gene Delivery to Diverse Mammalian Cells In Vitro and to Multiple Organs In Vivo

**DOI:** 10.3390/ijms27010389

**Published:** 2025-12-30

**Authors:** Min-Hsiu Wu, Song-Tay Lee, Tsung-Hsien Chang, Wei-Sheng Chao, Nan-Kai Lin, Shoa-Lin Lin

**Affiliations:** 1 Department of Medical Research, Chi Mei Medical Center, Tainan 710832, Taiwan; galenmhwu@yahoo.com; 2 Department of Biotechnology and Food Technology, Southern Taiwan University of Science and Technology, Tainan 71005, Taiwan; songtlee@stust.edu.tw (S.-T.L.); vcsavior15@gmail.com (W.-S.C.); wanderingdog@hotmail.com (N.-K.L.); 3 Department and Graduate Institute of Microbiology and Immunology, National Defense Medical University, Taipei 11490, Taiwan; cthvghks@gmail.com; 4 Division of Cardiology, Department of Internal Medicine, Yuan’s General Hospital, Kaohsiung, 162, Cheng-Kung First Road, Lingya District, Kaohsiung City 80249, Taiwan

**Keywords:** animal, baculovirus, gene delivery, gene therapy, recombinant baculovirus vector

## Abstract

Recombinant baculovirus vectors are recognized as effective gene delivery systems for mammalian cells in vitro. However, their application in vivo has been limited due to inactivation by the host’s complement system. We developed a recombinant baculoviral vector derived from Autographa californica multiple nucleopolyhedrovirus (AcMNPV), incorporating both CMV-IE and polyhedron promoter-driven green fluorescence protein (EGFP) (vAcMBac-CMV-IE-EGFP). We then evaluated the transduction efficiency and safety of vAcMBac-CMV-IE-EGFP at a high multiplicity of infection (MOI) across five distinct cell lines and in Sprague Dawley (SD) rats. In vitro, Sf9, HepG2, and Vero E6 cells showed high transduction rates (95.52 ± 4.86%, 80.53 ± 3.31%, and 80.87 ± 2.50%, respectively), significantly outperforming the other cell types tested, and cell viability remained largely unaffected even at an MOI of 1000. In vivo, EGFP expression was observed in the heart, liver, spleen, lungs, and kidneys of SD rats after tail vein injection. Direct injection of vAcMBac-CMV-IE-EGFP into the rat striatum also resulted in strong EGFP signals in neural tissues. These results demonstrate that a high-MOI baculovirus infection can serve as a remarkably efficient and versatile platform for gene delivery across diverse mammalian cell types as well as in various organs and neural tissues in animal models. This robust method might hold significant promise for future gene therapy applications.

## 1. Introduction

Baculoviruses, members of the *Baculoviridae* family, are best represented by Autographa californica multiple nuclear polyhedrosis virus (AcMNPV), an enveloped virus with a circular double-stranded DNA genome of 80–180 kb. The insect specificity of baculoviruses has led to their application as safe potential insecticides for use against forestry and agricultural pests [1]. This use has prompted an investigation into the possibility of baculovirus replication in vertebrate systems. Low levels of AcMNPV replication have been reported in Chinese hamster cell lines [2]. Although numerous studies have identified various mammalian cell types as being susceptible to baculovirus transduction, there is no evidence of viral replication, persistence, or cytopathic effects in these cells [3,4,5,6]. In 1995, Hofmann et al. created recombinant AcMNPV equipped with a cytomegalovirus (CMV) promoter to activate the truncated large T antigen of SV40 in human hepatocytes, thereby demonstrating baculovirus-mediated gene transfer into mammalian cells in vitro [7]. Since then, an increasing number of mammalian cell lines have been shown to support AcMNPV transduction [8,9,10,11,12]. These studies have encouraged the development of baculovirus as a potential gene delivery vector for both in vitro and in vivo gene therapy applications.

The restriction of in vivo delivery of genes by baculovirus vectors into mammalian cells via systemic administration has been attributed to two major factors: inactivation of the virus by the mammalian complement system [13,14,15,16,17] and reduced transgene expression efficiency due to induction of innate antiviral immune responses, such as interferons (IFNs) [18,19,20,21,22,23,24]. However, the use of baculovirus vectors in ex vivo applications and in immune-privileged sites—such as tissue-engineered cartilage and bone—has met with promising success [25].

The baculovirus-based gene expression method has been shown to ameliorate ischemia-reperfusion injury (IRI) in organs during transplantation procedures [26]. Moreover, recombinant baculoviruses have demonstrated the ability to penetrate mammalian kidneys and livers during cold preservation [27]. Baculoviruses’ potential as gene therapy vectors for brain cancer has also been evaluated [28]. In addition, the baculovirus expression vector has emerged as a promising platform for vaccine production, offering high safety, rapid scalability, and flexible product design [29]. Collectively, these studies highlight the broad applicability of baculovirus-based vectors.

Recently, several studies have shown that recombinant baculoviruses can mediate in vivo gene transfection. One such study demonstrated that the delivery of baculovirus to adventitial cells of rabbit carotid arteries via a collar-mediated approach was efficient, although transgene expression was transient. In this study, LacZ-baculoviruses were delivered using a silastic collar placed around the rabbit carotid arteries—a design that effectively avoids contact with blood components [30]. In subsequent studies, researchers used recombinant AcMNPV as a transfer vector for in vivo targeting of different organs, including the brain, eyes, skeletal muscles, cerebral cortex, and testes [31,32,33,34,35,36]. However, these studies relied on direct injection of recombinant baculoviruses into target organs to transfer genes. Whether baculovirus-based vectors can mediate gene expression in animal organs following intravenous administration remains unknown.

In this study, we first constructed a recombinant AcMNPV, designed as a dual-expression vector containing both the CMV immediate-early promoter (CMV-IE, Pcmv) and the polyhedron promoter (Pph). We then optimized key parameters to enhance baculovirus transduction efficiency across various mammalian cell types. Finally, we evaluated in vivo baculovirus-mediated gene delivery by administering the vector via tail vein injection into Sprague Dawley (SD) rats, assessing enhanced green fluorescent protein (EGFP) expression in the heart, liver, spleen, lungs, and kidneys. In parallel, we performed stereotaxic injections into the striata of SD rats to monitor EGFP expression in neural tissues.

## 2. Results

### 2.1. Construction of a Dual-Promoter Recombinant AcMNPV Expression Vector

**Figure 1:**
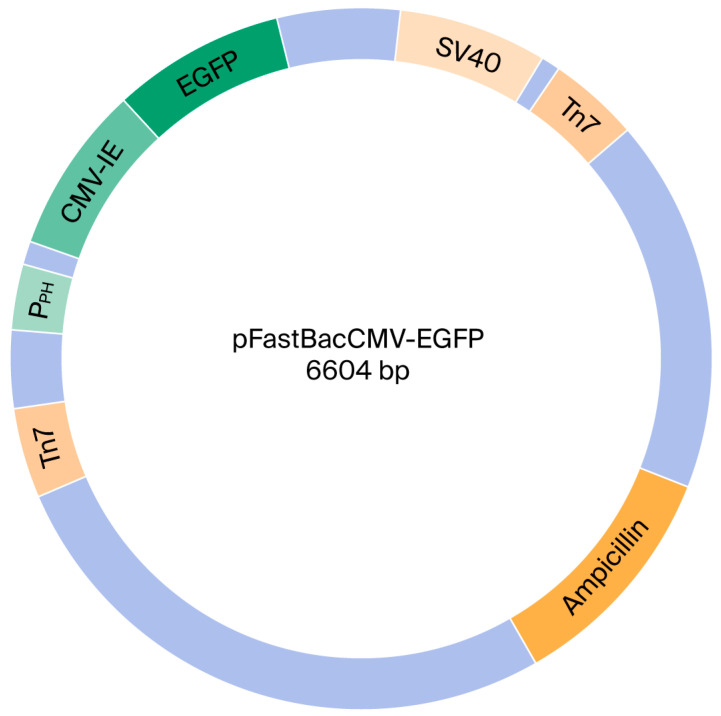
Plasmid map of recombinant baculoviral vector.

**Figure 2:**
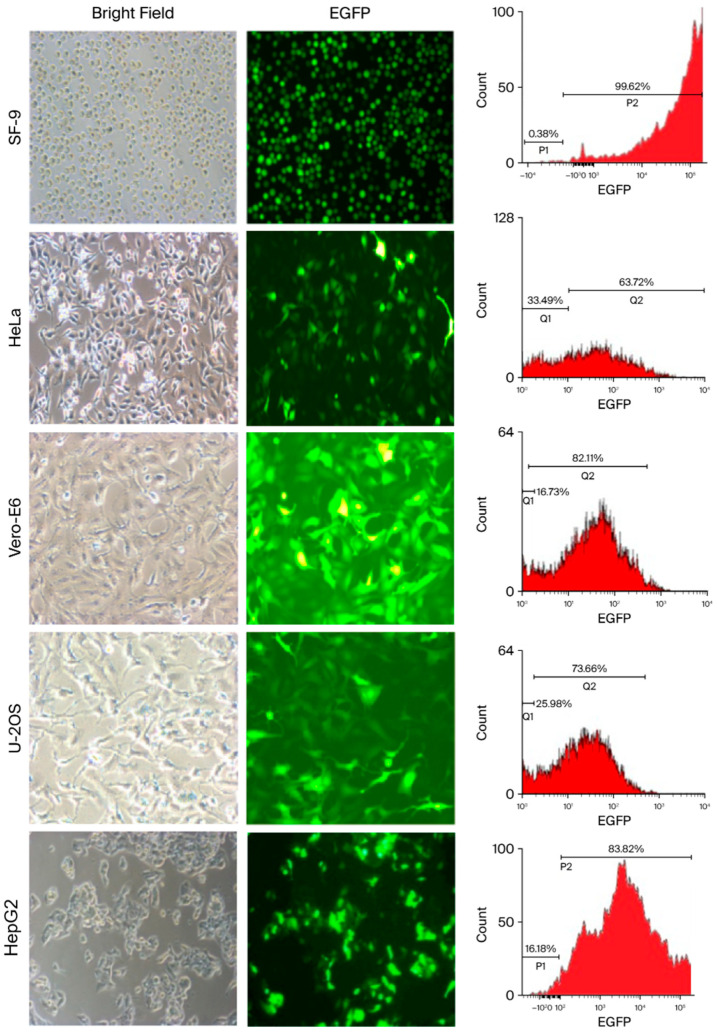
Light and fluorescence microscopy and flow cytometry images demonstrating the efficiency of transduction of vAcMBac-CMV-IE-EGFP into insect sf9 cells and mammalian cell lines, including HeLa, Vero E6, U-2 OS, and HepG2.

A recombinant AcMNPV containing dual promoters was created and designated vAcMBac-CMV-IE-EGFP. This construct harbors an EGFP expression cassette whose activity is driven by both the CMV-IE and Pph promoters (Figure 1), as detailed in the Materials and Methods Section. The ability of vAcMBac-CMV-IE-EGFP to infect both insect cells and mammalian cells was evaluated by assessing EGFP expression using fluorescence microscopy and flow cytometry after 48 h of viral infection at a multiplicity of infection (MOI) of 500. The transduction efficiency in the insect cell line Sf-9 reached 95.52 ± 4.86%, while the efficiencies achieved in the mammalian cell lines HeLa, Vero E6, U-2 OS, and HepG2 were 60.87 ± 2.59%, 80.87 ± 2.50%, 70.14 ± 1.70%, and 80.53 ± 3.31%, respectively (Figure 2 and Table 1). The observation of over 50% transduction efficiency in Sf-9 cells and in most tested mammalian cell lines indicates that both the polyhedron (Pph) and CMV-IE promoters in the viral construct were functionally active.

### 2.2. High-Dose Baculovirus Transduction Exhibits Minimal Cytotoxicity in Mammalian Cells

**Figure 3:**
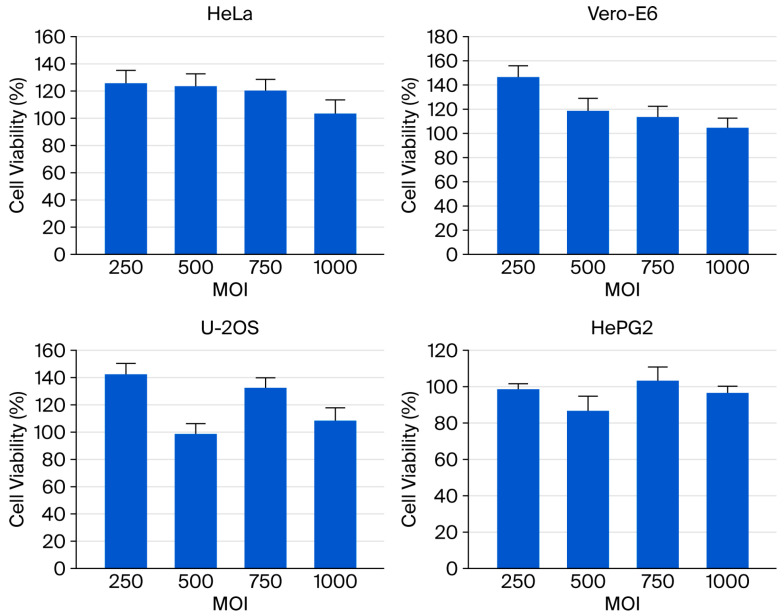
The viability of mammalian cells—including HeLa, Vero E6, U-2 OS, H9c2, and HepG2—transduced with vAcMBac-CMV-IE-EGFP at MOIs of 250, 500, 750, and 1000 was assessed using an MTT assay at 48 h post-transduction (*n* = 6).

Although baculovirus’s inherent inability to replicate and low cytotoxicity in mammalian cells have been previously reported, the effects of extremely high viral dosages on transduction have not been thoroughly examined. In this study, the cytotoxicity of the baculoviral vector was assessed in four mammalian cell lines at MOI values ranging from 250 to 1000 using the MTT assay. After 48 h of viral infection, no obvious cytopathic effects were observed in any of the tested cells, which maintained viability above 95% even at the highest MOI tested (Figure 3). These findings indicate that recombinant baculovirus infection in mammalian cells exhibits minimal cytotoxicity, supporting its potential as a safe gene delivery method.

### 2.3. Temporal Dynamics of Baculovirus-Mediated Transduction Efficiency

The transduction efficiency of baculovirus is typically assessed by quantifying EGFP-expressing cells using fluorescence microscopy at 48 h post-infection (p.i.), when infected cells generate sufficient fluorescence intensity for detection. However, vAcMBac-CMV-IE-EGFP is expected to be internalized by cells at earlier time points. To investigate this, HeLa cells were inoculated with or without vAcMBac-CMV-IE-EGFP (MOI = 500), and the EGFP-expressing cells were analyzed at 12, 24, and 48 h p.i. using both flow cytometry and fluorescence microscopy.

**Figure 4:**
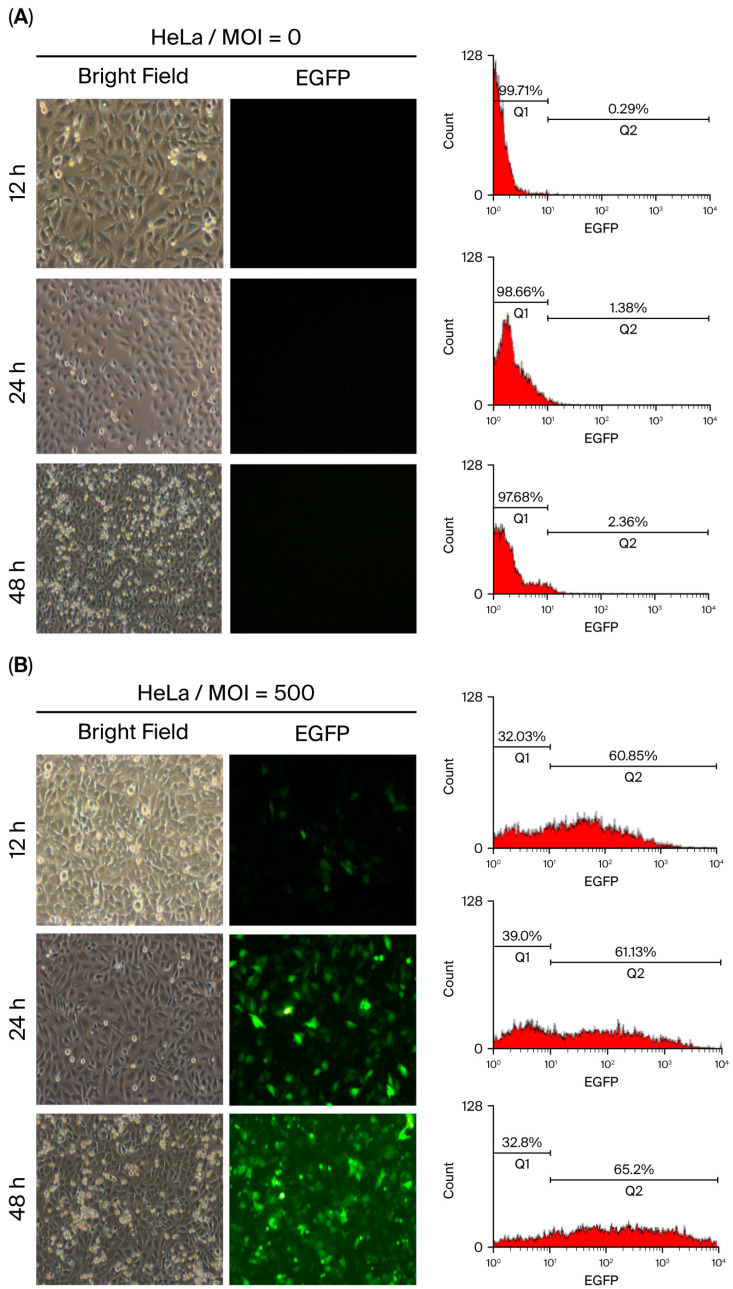
Transduction efficiency of HeLa cells following infection with vAcMBac-CMV-IE-EGFP at an MOI of 0 (**A**) or 500 (**B**). EGFP-expressing cells were evaluated via fluorescence microscopy and flow cytometry at 12, 24, and 48 h post-infection. EGFP expression was detectable via flow cytometry as early as 12 h post-infection, whereas fluorescence microscopy revealed a minimal signal at this time point.

Uninfected HeLa cells served as negative controls and showed no EGFP signal (Figure 4A). Flow cytometry analysis revealed that approximately 58.87 ± 1.83% of inoculated cells expressed EGFP at 12 h p.i., with fluorescence intensities ranging from 10^1^ to 10^4^. However, only a few EGFP-expressing cells were visible via fluorescence microscopy at this time point. At 24 h p.i., 59.40 ± 1.79% of cells expressed EGFP, and at 48 h p.i., the proportion increased to 62.86 ± 1.43%, with both having similar fluorescence intensity ranges (Figure 4B and Table 2). These results indicate a progressive increase in EGFP-expressing cells over time. While fluorescence microscopy observation at 48 h p.i. is sufficient to evaluate baculovirus transduction efficiency, flow cytometry enables earlier detection at 12 h p.i. with greater sensitivity, suggesting that flow cytometry may be more effective than fluorescence microscopy for early assessment of transduction efficiency.

### 2.4. Optimal Parameter for Assessing the Transduction Efficiency of Baculovirus

**Figure 5:**
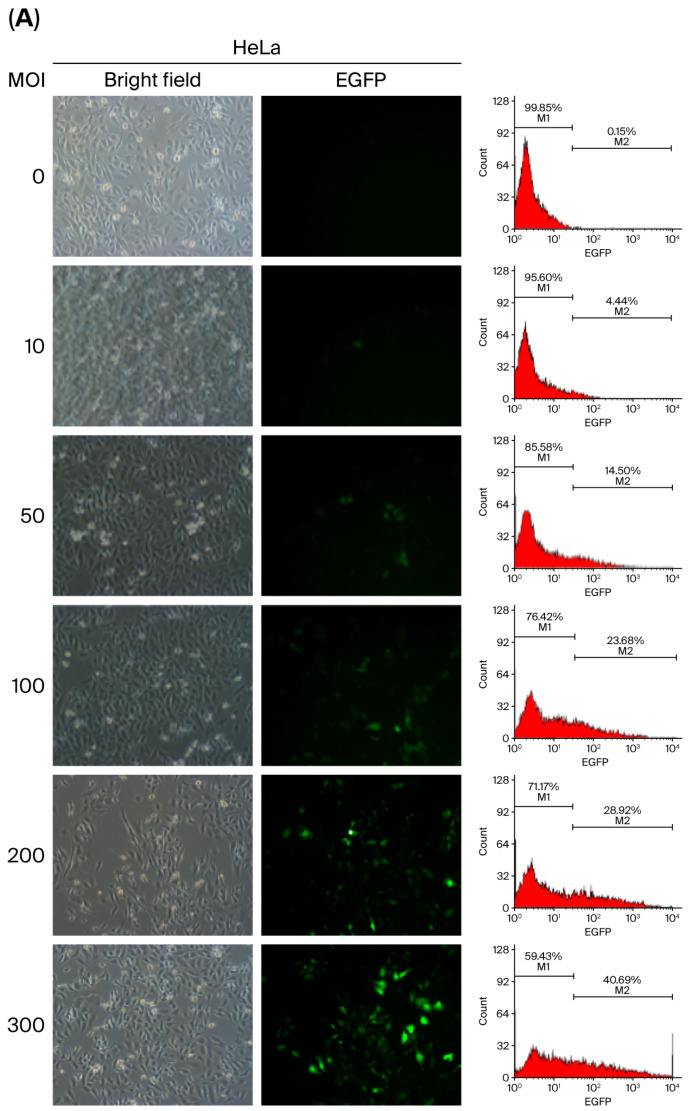

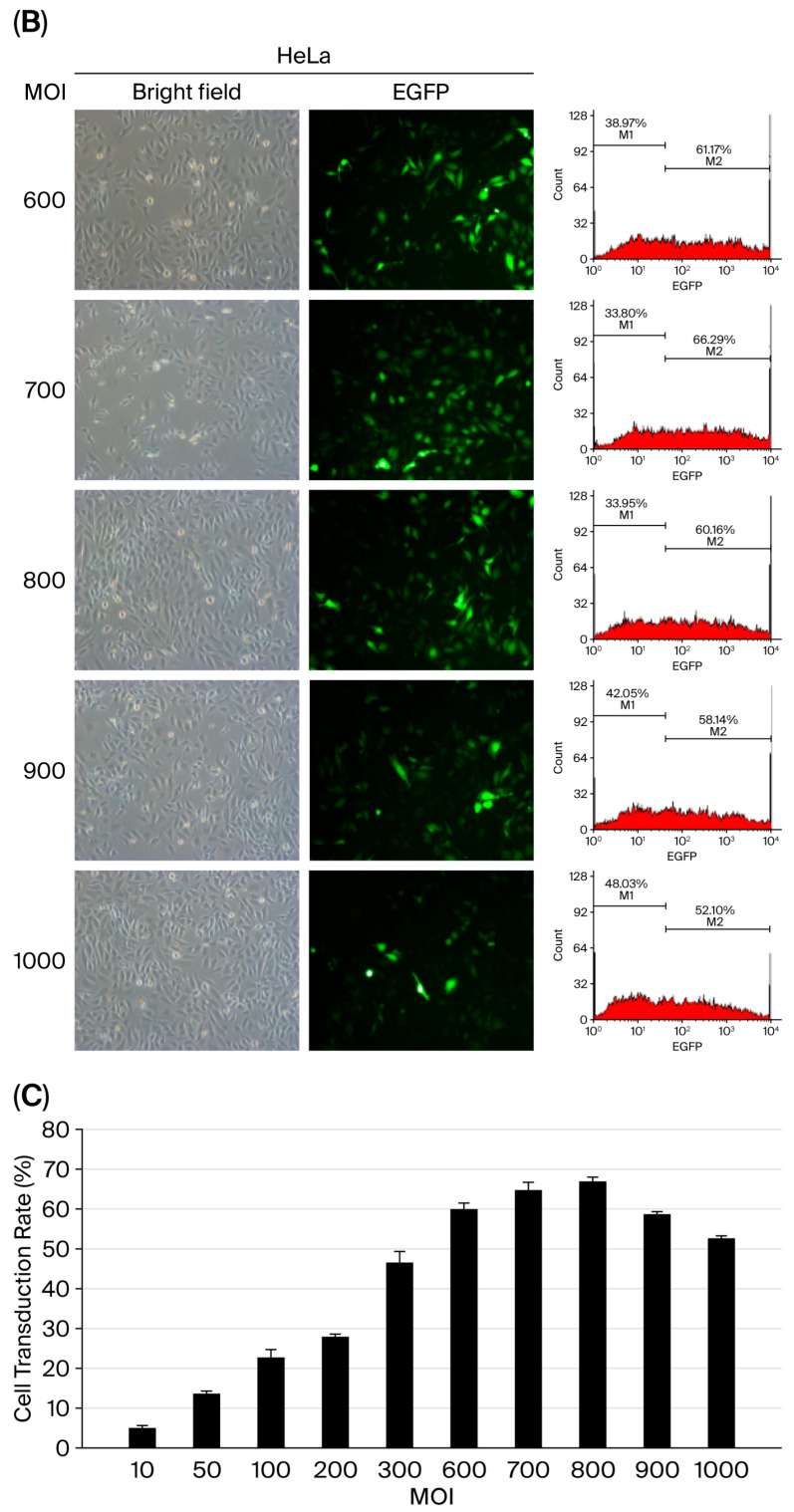
The transduction efficiency of HeLa cells (5 × 10^5^) infected with vAcMBac-CMV-IE-EGFP was assessed at various viral dosages: MOI = 0–300 (**A**) and MOI = 600–1000 (**B**). After a 2 h incubation period, EGFP-expressing cells were detected via fluorescence microscopy (left panels) and flow cytometry (right panels) at 48 h post-infection. (**C**) The proportion of EGFP-expressing cells from three independent flow cytometry analyses was quantified. Transduction efficiency progressively increased as the MOI was raised from 10 to 700.

The transduction efficiency of baculoviral vectors is influenced by inoculation parameters such as viral load and exposure duration. To determine the optimal conditions for baculovirus-mediated gene delivery in mammalian cells, we used HeLa cells due to their intermediate transduction efficiency (60.87 ± 2.59%) at an MOI of 500, as previously described in Figure 2. A range of viral doses (MOI 10 to 1000) was tested, and transduction efficiency was assessed by quantifying EGFP-positive cells using flow cytometry and fluorescence microscopy at 48 h p.i. The results showed a progressive increase in transduction efficiency with an increasing MOI (Figure 5A,B), reaching a peak of 64.41% at an MOI of 700. However, efficiency declined to 51.35% at MOI 1000 (Figure 5C), suggesting there is a threshold beyond which higher viral loads may reduce transduction performance.

**Figure 6:**
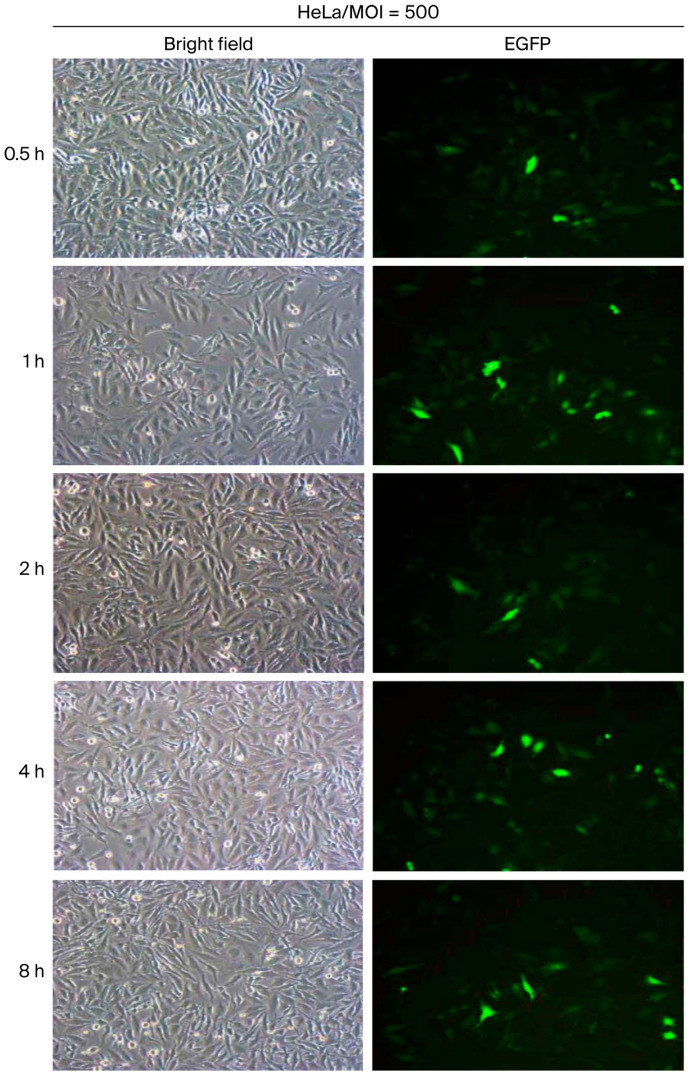
The transduction efficiency of HeLa cells infected with vAcMBac-CMV-IE-EGFP at an MOI of 500 was evaluated. Cells were gently rocked for 0.5, 1, 2, 4, and 8 h to determine the optimal incubation period.

Prolonging the virus incubation period is generally expected to enhance transduction efficiency. However, the optimal incubation duration at a given viral dosage remains to be defined. In this study, HeLa cells were inoculated with vAcMBac-CMV-IE-EGFP at a multiplicity of infection (MOI) of 500 and incubated with gentle rocking for 0.5, 1, 2, 4, or 8 h to determine the optimal exposure time. The transduction efficiency was 44.8% after 0.5 h of incubation and increased to 50.4%, 60.7%, and 60.3% following 1, 2, and 4 h of incubation, respectively. However, extending the incubation period to 8 h resulted in a slight decline in efficiency to 56.2% (Figure 6 and Table 3). According to these results, transduction efficiency in mammalian cells, as evaluated in HeLa cells, was optimal at an MOI of 600–800 with a 2–4 h incubation period in which gentle rocking was employed.

### 2.5. Recombinant AcMNPV as an In Vivo Gene Delivery Vector Targeting Mammalian Organs

**Figure 7:**
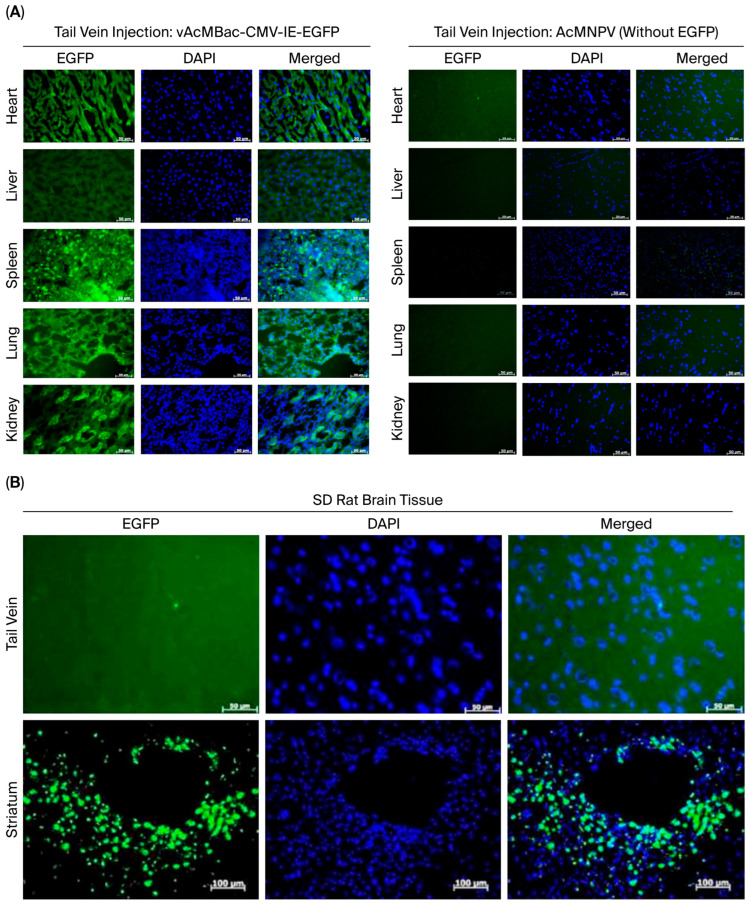
Microscopic images of EGFP gene expression in tissue sections from SD rats inoculated with vAcMBac-CMV-IE-EGFP (5 × 10^7^ PFU/mL): (**A**) **Left panels**: SD rats were injected via the tail vein with 3.5 mL of vAcMBac-CMV-IE-EGFP and sacrificed 72 h later. EGFP protein expression was detected in the heart, liver, spleen, lungs, and kidneys. No EGFP expression was observed in the cerebral cortex following tail vein injection. **Right panels**: The control group was subjected to the same procedures as the experimental group, except they were inoculated with AcMNPV without harboring the EGFP gene. EGFP protein expression was not detected in the heart, liver, spleen, lungs, or kidneys. Scale bar: 50 μM. (**B**) EGFP protein expression was detected in the striatum following direct injection of 20 μL of vAcMBac-CMV-IE-EGFP into this region (scale bar: 100 μM). However, no EGFP expression was observed in the striatum when the recombinant baculovirus was administered via tail vein injection (scale bar: 20 μM). EGFP, green fluorescence protein; DAPI, 4′,6-diamidino-2-phenylindole.

We demonstrated that several mammalian cell lines inoculated with a high viral dose of up to MOI 1000 exhibited no evidence of cytopathic effects (Figure 3). To assess whether a high-MOI infection could mediate transgene expression in vivo, Sprague Dawley (SD) rats were intravenously injected via the tail vein with 3.5 mL of vAcMBac-CMV-IE-EGFP at a concentration of 5 × 10^7^ PFU/mL. At 72 h p.i., the animals were sacrificed, and EGFP expression was evaluated in various organs. The control Sprague Dawley (SD) rats underwent the same procedures as the experimental group but were inoculated with AcMNPV lacking the EGFP gene. EGFP expression was clearly detected in the heart, liver, spleen, lungs, and kidneys of SD rats inoculated with vAcMBac-CMV-IE-EGFP, whereas no EGFP signal was observed in rats that received AcMNPV without the EGFP gene (Figure 7A). We did not observe an EGFP signal in the brains of SD rats after tail vein inoculation of vAcMBac-CMV-IE-EGFP. However, direct injection of 20 μL of vAcMBac-CMV-IE-EGFP into the striatum (a part of the basal ganglia) successfully transduced neural cells in vivo (Figure 7B). These data suggest that recombinant baculovirus can be used for transgene delivery in vivo.

## 3. Discussion

Gene therapy has advanced to the point where it can be used to treat a variety of illnesses, including genetic deficiencies, cancer, and rare diseases, and it can even be used in wound repair. Its success largely depends on the development of suitable delivery vehicles that can efficiently and safely introduce foreign genes into mammalian cells. Both viral and nonviral vectors have been employed in this field [37], with viral vectors generally considered the more effective platforms for gene delivery [38,39]. Several viral vectors, including adeno-associated vectors (AAVs), lentivirus, and retrovirus, have been approved by the US Food and Drug Administration (FDA) for gene therapy implementation [40]. However, AAV vectors face challenges in large-scale production and carry risks of toxicity and inflammation. Lentiviral and retroviral vectors also suffer from limited cloning capacity, and their genomes may integrate into host DNA, raising the possibility of insertional mutagenesis. In addition, the replication properties of retroviruses have raised safety concerns regarding oncogenesis, including virus-induced T-cell lymphomas. These disadvantages restrict their broader application [41].

Engineered recombinant baculoviruses equipped with mammalian cell-active promoters can enter a broad range of mammalian cells and transiently express foreign genes without undergoing viral replication. Their inability to replicate in mammalian cells, combined with their low cytotoxicity, makes them relatively safe, scalable, and cost-effective, thereby establishing baculoviruses as valuable vectors for gene therapy [7,8,10,11].

In this study, we generated a recombinant AcMNPV carrying the shuttle promoters CMV-IE (Pcmv) and polyhedron (Pph) to potentiate EGFP expression. We hypothesized that vAcMBac-CMV-IE-EGFP expressed EGFP in mammalian cells under the control of the CMV-IE promoter/enhancer, while expression in insect cells was driven by the polyhedron promoter (Pph). We demonstrated that this recombinant baculovirus, vAcMBac-CMV-IE-EGFP, mediated EGFP expression in both insect and mammalian cells. In the permissive insect cell line Sf-9, transduction efficiency approached 96%, whereas in four non-permissive mammalian cell lines (Vero E6, HeLa, U-2 OS, and HepG2), effective transduction rates ranged from 61% to 81%. These results confirmed robust EGFP expression in both permissive and non-permissive mammalian cells, highlighting the therapeutic potential of baculovirus, although efficiency varied by cell type (Figure 2).

Regarding the therapeutic potential of baculoviral vectors, safety is an important concern. Many researchers have reported that baculoviruses replicate only in insects and not in mammalian cells [42,43]. In this study, all tested mammalian cells infected with an extremely high dosage of vAcMBac-CMV-IE-EGFP at an MOI of up to 1000 showed no evidence of cytopathic effects (Figure 3). Our findings indicate that vAcMBac-CMV-IE-EGFP transduction in mammalian cells is safe even at extremely high concentrations, a finding consistent with previous reports [44].

The mechanisms underlying baculoviruses’ binding with and entry into mammalian cells remain incompletely understood. Several studies have investigated potential receptors on mammalian cells and viral molecules that facilitate baculoviruses’ attachment to the cell membrane. It has been reported that baculovirus gp64 and cell-surface heparan sulfate are required for viral entry [45,46,47], with GP64 binding to heparan sulfate proteoglycans (HSPGs) [48]. These findings have prompted efforts to modify baculoviral surface molecules, such as incorporating vesicular stomatitis virus envelope G protein (VSVG) or increasing gp64 expression, to enhance gene transfer efficiency [47,49]. Following attachment to cell-surface molecules, baculoviruses are internalized via lipid rafts [45]. Infection of insect cells and transduction of mammalian cells by baculoviruses have been extensively shown to occur primarily through endocytosis [49,50,51]. However, Dong et al. demonstrated that AcMNPV enters Sf9 cells through direct fusion at low pH, and that acidic conditions can significantly enhance baculovirus entry into mammalian cells, thereby increasing transduction efficiency [52,53,54].

The transduction efficiency of baculoviruses can be improved by optimizing transduction conditions. A previous study reported that when HeLa cells were transduced with baculovirus, incubation for 4 h at 25 °C in Dulbecco’s phosphate-buffered saline (D-PBS) resulted in higher expression of the reporter protein GFP [53].

Although baculovirus infection has been shown to induce antiviral responses in mammalian cells, we hypothesized that administering a high dosage of recombinant AcMNPV could overcome inactivation mediated by innate immune mechanisms, such as the complement system and interferons (IFNs). Our in vivo data demonstrated that Sprague Dawley (SD) rats inoculated via the tail vein with 3.5 mL of vAcMBac-CMV-IE-EGFP at a concentration of 5 × 10^7^ PFU/mL exhibited clear EGFP expression in the heart, liver, spleen, lungs, and kidneys but not in the brain. These findings suggest that systemic delivery of recombinant baculovirus vectors at high, non-lethal doses can allow effective transgene expression in vivo, providing evidence of their potential utility in gene therapy applications.

There are two limitations that should be mentioned. Firstly, although this study demonstrated that EGFP protein expression was detected in the heart, liver, spleen, lungs, and kidneys tissues from SD rats inoculated with vAcMBac-CMV-IE-EGFP (Figure 7A, left panels), it did not demonstrate the EGFP protein expression in those without harboring the EGFP gene (Figure 7A, right panels). However, we have not assessed the RT-PCR, Western blot, ELISA, or fluorescence assay in a plate reader of the rat tissues. This study has not provided more supportive data on EGFP gene expression analysis, which is a limitation of this study. Secondly, we carried out a comparison of EGFP protein expression in the striatum by direct injection versus tail vein injection of vAcMBac-CMV-IE-EGFP. This study only performed a qualitative comparison of the fluorescence imaging without any quantitative comparison between these two experiments. This was another limitation.

## 4. Materials and Methods

### 4.1. Cells

The insect cell line Sf-9, isolated from ovarian tissue of Spodoptera frugiperda, was purchased from Bioresource Collection and Research Center, Hsinchu, Taiwan (catalog number CCRC60011), and cultured in TC-100 insect cell culture medium at 26 °C with 10% heat-inactivated fetal bovine serum (FBS) (Gibco). The mammalian cell lines Vero-E6 and U-2 OS were provided by Professor Yu-Chan Chao from the Institute of Molecular Biology, Academia Sinica, Nankang, Taipei, Taiwan. The HeLa and HepG2 cell lines were provided by Chi-Mei Medical Center, Tainan, Taiwan. Vero-E6 cells isolated from African green monkey kidneys were incubated in Minimum Essential Medium (MEM) with 2 mM L-glutamine, 1.5 g/L sodium bicarbonate, 1% sodium bicarbonate, and 0.1 mM nonessential amino acids, supplemented with 10% (*v*/*v*) FBS. U-2 OS, a human osteogenic sarcoma cell line, was incubated in McCoy 5A medium with 1.5 mM L-glutamine and 10% (*v*/*v*) FBS. HeLa cells from a human cervical epithelioid carcinoma were maintained at 37 °C in MEM with 2 mM L-glutamine, 1.5 g/L of sodium bicarbonate, 0.1 mM non-essential amino acids, and 1.0 mM sodium pyruvate, supplemented with 10% (*v*/*v*) FBS. HepG2, a human hepatocellular carcinoma cell line, was maintained in Dulbecco’s Modified Eagle Medium (DMEM) supplemented with 2 mM L-glutamine, 10 mM HEPES, and 10% (*v*/*v*) FBS. All mammalian cell lines were maintained at 37 °C in a 5% C02-95% air environment.

### 4.2. Plasmids

pAtEG containing an insert of the EGFP gene was kindly provided by Professor Yu-Chan Chao from the Institute of Molecular Biology, Academia Sinica, Nankang, Taipei, Taiwan. pFastBac was purchased from Life Technologies, Grand Island, NY, USA.

### 4.3. Construction of Recombinant AcMNPV with Dual Promoters Harboring EGFP Expression Cassette

We modified the transvector pFastBac1, one component of Bac-to-Bac^®^ Baculovirus Expression System (Life Technologies Corporation, Grand Island, NY, USA), for the construction of recombinant AcMNPV via insertion of the CMV-IE (Pcmv) promoter in the anterior part of the multiple cloning sites through BamHI and EcoRI restriction endonuclease enzyme sites. The CMV-IE fragment was amplified by PCR from plasmid pAtEG. The primer sequences subjected to amplification of CMV-IE fragments were as follows: forward primer, 5′CGAGGATCCACAAACTGGAAATGTCTATC3′, and reverse primer, 5′GCTGAATTCGGTATATCTCCTTTGATTGT3′. The generated transvector pFastBac1-CMV-IE was equipped with dual promoters, Pph and Pcmv, to express foreign genes in insect Sf-9 cells and mammalian cells, respectively. The posterior parts of the multiple cloning sites of pFastBac1-CMV-IE were kept for cloning foreign genes. For the construction of recombinant AcMNPVs harboring the enhanced green fluorescence protein (EGFP) expression cassette, the EGFP coding region was obtained via PCR from pAtEG and then cloned into the pFastBac1-CMV-IE between Spe I and Xho I restriction endonuclease enzyme sites to generate pFastBac1-CMV-IE-EGFP. The primer sequences for amplification of the EGFP gene were as follows: forward primer, 5′TATACTAGTCATGGTGAGCAAGGGCGAGG3′, and reverse primer, 5′CGTCTCGAGTTACTTGTACAGCTCGTCCA’.

Then, pFastBac1-CMV-IE-EGFP was transformed into *E. coli* component, the MAX Efficiency^®^ DH10Bac™, of Bac-to-Bac^®^ Baculovirus Expression System. DH10Bac™ was internalized as a modified AcMNPV (bacmid), a large extra genome of *E. coli* cells, and a helper plasmid that expresses transposase. The sequences of Tn7 L and Tn7 R transposition sites were cloned into both pFastBac1 and bacmid. Furthermore, a lacZ (b-galactosidase gene) was designed to be located between Tn7 L and Tn7 R in the bacmid. When the pFastBac1-CMV-IE-EGFP was transformed into the DH10Bac, the transposase served to replace the EGFP gene expression cassette from pFastBac1-CMV-IE-EGFP with the lacZ gene located in the bacmid. Ideally, the successful recombinant bacmid should lose lacZ gene function and be identified by blue and white colony screening. The white colony, which harbors the recombinant bacmid, was selected and cultured for further purification. The purified bacmid DNA, i.e., bFastBac-CMV-IE-EGFP harboring the EGFP gene expression cassette, was further transfected into insect cells, Sf-9, to replicate recombinant AcMNPV, designated as vAcMBac-CMV-IE-EGFP.

### 4.4. Baculovirus Production in Insect Cells and Titration

Wild-type AcMNPV and its derived recombinant viruses were generated and propagated in Sf-9 cells according to the standard protocols described by O’Reilly et al. [55]. The titers of the virus clones were determined via both quantitative PCR (qPCR) and the 50% tissue culture infective dose (TCID50) [56].

### 4.5. In Vitro Transduction of Insect and Mammalian Cells with Recombinant AcMNPV

A monolayer of cells was cultured in a 6-well plate in a matched medium of each cell line for 12 h before viral inoculation. Then, the culture medium was removed and replaced with 1000 μL of fresh FBS-free medium containing recombinant AcMNPV, vAcMBac-CMV-IE-EGFP, at a multiplicity of infection (MOI) ranging from 250 to 1000. After an adsorption period with gentle rocking, ranging from 0.5 h to 8 h, the inoculum was removed, and cells were washed with phosphate-buffered saline (PBS) with a pH of 7.4. Then, a fresh medium with FBS was added. The insect cells were incubated at 26 °C, and mammalian cells were cultured at 37 °C, with 5% CO_2_.

### 4.6. Flow Cytometry

The cells were examined for enhanced green fluorescent protein (EGFP) expression at 48 h post-infection (p.i.) via fluorescence microscopy and flow cytometry. The cells were then harvested with trypsin, washed with PBS twice, and resuspended in 1 mL of DPBS. The cells were collected and analyzed using an LSR II flow cytometer (Becton-Dickinson, San Jose, CA, USA). The EGFP proteins within the cells were excited at 488 nm and detected at 525 nm. About 10^4^ cells with three repeats were collected per specimen. Cells without virus transduction were used as the negative control. Transduction efficiency was obtained as a percentage: the number of EGFP-positive cells over the number of total cells collected. The level of EGFP expression was measured as the mean fluorescence intensity multiplied by the total cell number for each sample. Data are expressed as means ± standard deviation (SD).

### 4.7. 3-(4,5-Dimethylthiazol-2-Yl)-2,5-Diphenyltetrazolium Bromide (MTT) Assay

Cell viability subject to AcMNPV transduction was examined via MTT assay. An appropriate number of cells at about 50% confluence were cultured in a 96-well plate in a suitable medium for 12 h. Then, viral transduction, as described above, was performed. At 72 h after inoculation, 20 μL of MTT solution (Merck, Darmstadt, Germany) (5 mg/mL PBS) was added to each well, and the plate was incubated at 37 °C for 4 h. After medium removal, 200 μL of DMSO was added to each well, and the plate was gently shaken for 5 min. Absorbance was determined at 540 nm. Quadruplicate wells were applied to each MOI for a specific transduction. The 0.002% (*v*/*v*) DMSO (vehicle)-treated non-transfected cells were employed as the control.

### 4.8. In Vivo Transduction of SD Rats with Recombinant AcMNPV

The in vivo transduction of vAcMBac-CMV-IE-EGFP was conducted with male Sprague Dawley (SD) rats weighing 350–400 g via either tail vein or striatum injection of the virus with an infectious titer of 5 × 10^7^ PFU/mL. For tail vein injection, SD rats in the study group (*n* = 6) were injected with vAcMBac-CMV-IE-EGFP, which harbors an EGFP expression cassette. The rats in the control group (*n* = 6) were injected with AcMNPV, which does not harbor the EGFP gene. The SD rats without anesthesia were injected with 3.5 mL of AcMNPV via tail vein inoculation. For striatum infection (*n* = 6), 20 μL vAcMBac-CMV-IE-EGFP was injected into the striatum of SD rats, which were anesthetized with Urethane (100 mg/kg). After 72 h p.i., the infected rats were anesthetized with urethane (100 mg/kg) and sacrificed. The rats were immediately transferred to a sterile bench for cardiac perfusion with physiological saline to remove blood, followed by fixation with 4% paraformaldehyde. Brain, heart, lung, liver, spleen, and kidney tissues were carefully dissected under sterile conditions and placed in pre-cooled PBS. Fragile organs such as the brain and lungs were handled gently to avoid structural damage, while the liver and kidney samples were trimmed into standardized blocks (approximately 1 cm × 1 cm × 0.3 cm). All tissues were rinsed in PBS for 5 min to remove residual blood and fluids and then blotted dry with sterile filter paper. Processed samples were embedded in OCT compound and frozen for 15–20 min until solidified, sectioned at 5–10 µm thickness (5 µm for brain and 8 µm for liver and kidney), and subjected to EGFP immunofluorescence and nuclear DAPI staining. Fluorescence signals were observed directly under a fluorescence microscope using excitation at ~488 nm to detect EGFP expression. All animal experiments were approved by the Institutional Animal Care and Use Committee (IACUC) of Southern Taiwan University.

### 4.9. Statistical Analysis

Statistical analysis of flow cytometry data between the control group and the vAc-MBac-CMV-IE-EGFP transduction group was performed using Student’s *t*-test, with differences considered significant at *p* < 0.05.

## 5. Conclusions

We developed a high-titer, dual-promoter recombinant baculovirus (vAcMBac-CMV-IE-EGFP) capable of efficient and safe gene delivery. The use of high MOI baculovirus resulted in high transduction efficiencies in vitro, with over 80% in HepG2 and Vero E6 cells, and low cytotoxicity even at an MOI of 1000. In vivo, tail vein injection in rats resulted in robust EGFP expression across multiple organs, demonstrating that systemic delivery at high, non-lethal doses can overcome immune inactivation. Moreover, successful localized delivery to the rat striatum highlights this method’s potential for targeted applications. These findings establish this high MOI baculovirus infection as a versatile and promising platform for both systemic and tissue-specific gene therapy.

## Figures and Tables

**Table 1:** Transduction efficiency for insect and mammalian cells *.

	Transduction Efficiency (%)		
Cell Type	MOI = 0	MOI = 500	*p*-Value
Sf-9	0.25 ± 0.09	95.52 ± 4.86	0.00084
HeLa	0.18 ± 0.05	60.87 ± 2.59	0.0006
Vero-E6	0.32 ± 0.04	80.87 ± 2.50	0.00032
U-2 OS	2.12 ± 0.32	70.14 ± 1.70	0.0002
HepG2	1.83 ± 0.21	80.53 ± 3.31	0.00057

* The transduction efficiency for cells infected with vAcMBac-CMV-IE-EGFP at a multiplicity of infection (MOI) of 500 was evaluated after 48 h.

**Table 2:** The transduction efficiency of HeLa cells *.

	Transduction Efficiency (%)		
Infection Period (h)	MOI = 0	MOI = 500	*p*-Value
12	0.30 ± 0.03	58.87 ± 1.83	0.00033
24	1.23 ± 0.15	59.40 ± 1.79	0.00028
48	2.38 ± 0.06	62.86 ± 1.43	0.00017

* HeLa cells infected with vAcMBac-CMV-IE-EGFP at an MOI of 500 were assessed for transduction efficiency after 12, 24, and 48 h.

**Table 3:** Assessment of the effect of viral incubation periods on transduction efficiency in HeLa cells *.

	Transduction Efficiency (%)		
Incubation Period (h)	MOI = 0	MOI = 500	*p*-Value ^#^
0.5	0.12 ± 0.06	44.84 ± 3.76	0.00226
1.0	0.20 ± 0.17	50.42 ± 3.45	0.00151
2.0	0.12 ± 0.05	60.72 ± 7.37	0.00495
4.0	0.19 ± 0.06	60.34 ± 0.59	0.00004
8.0	0.15 ± 0.09	56.18 ± 9.12	0.00855

* Transduction efficiency for HeLa cells infected with vAcMBac-CMV-IE-EGFP at MOI 500, assessed in different viral incubation periods during which rocking was applied. # Student’s *t*-test was performed to compare data between two groups, and differences were considered significant at *p* < 0.05.

## Data Availability

The original contributions presented in this study are included in the article. Further inquiries can be directed to the corresponding authors.
